# The identification of QTLs and candidate genes associated with VW resistance of *Gossypium hirsutum* utilizing BSA-seq and QTL mapping

**DOI:** 10.1186/s12870-026-08833-y

**Published:** 2026-05-07

**Authors:** Juan Wang, Liang Zhao, Jianguang Liu, Jichong Chen, Xin Wang, Chengguang Dong, Jun Zhao

**Affiliations:** 1 Cotton Research Institute, Xinjiang Academy of Agricultural and Reclamation Science, Northwest Inland Region Key Laboratory of Cotton Biology and Genetic Breeding, Ministry of Agriculture and Rural Affairs, Shihezi, 832000 China; 2 Institute of Industrial Crops, Jiangsu Academy of Agricultural Sciences, Key Laboratory of Cotton and Rapeseed, Ministry of Agriculture and Rural Affairs, Nanjing, 210014 China; 3 Xinjiang Key Laboratory for Crop Gene Editing and Germplasm Innovation, Institute of Western Agricultural of CAAS, Changji, 831100 Xinjiang China

**Keywords:** Upland cotton, Verticillium wilt, Bulk segregant analysis (BSA), Quantitative trait loci (QTLs), Virus-induced gene silencing (VIGS).

## Abstract

**Background:**

*Verticillium wilt* (VW), a soil-borne fungal disease caused by *Verticillium dahliae*, causes severe yield losses in cotton production in China. Due to the scarcity of highly resistant upland cotton germplasm, the genetic improvement of VW resistance has progressed slowly.

**Results:**

In this study, we combined QTL mapping and bulked segregant analysis sequencing (BSA-seq) in an F₂ population derived from a cross between the resistant parent JM122 and the susceptible parent XLZ 4 and identified two stable VW-resistance QTLs. The QTL qVW-A05, located on chromosome A05, accounted for an average of 8.5% of the phenotypic variation; qVW-D07, on chromosome D07, explained an average of 12.3% of the phenotypic variation. Comparative analysis revealed that both QTLs overlapped with known VW-resistance hotspots. Association analysis in a natural population of 468 cotton accessions showed that genotypes carrying the same allele as the resistant parent JM122 at the linked markers exhibited significantly higher VW resistance. Based on expression profiles and sequence variation, 25 candidate genes from the two QTL regions were selected for functional validation using virus-induced gene silencing (VIGS). Silencing of *GH_A05G0294* (encoding TRX1), *GH_A05G0389* (encoding an Fd-like protein), *GH_D07G0616* (encoding MSR1) and *GH_D07G0638* (encoding a calcium-sensing receptor, CAS) significantly reduced VW resistance in the resistant parent JM122. Notably, silencing of *GH_D07G0638* (encoding a calcium-sensing receptor, CAS) in the susceptible parent XLZ 4 markedly enhanced its resistance, indicating that *GhCAS* may act as a critical susceptibility factor.

**Conclusion:**

Two stable VW-resistance QTLs (qVW-A05 and qVW-D07) and their linked markers were identified, providing valuable tools for marker-assisted selection in cotton breeding. Furthermore, four candidate genes which played key roles in VW resistance were validated, among which *GhCAS* was revealed for the first time as a potential susceptibility factor. These findings offer new genetic resources and theoretical insights for elucidating the cotton–*V. dahlia* interaction and developing resistant cultivars.

**Supplementary Information:**

The online version contains supplementary material available at 10.1186/s12870-026-08833-y.

## Introduction

Cotton (*Gossypium* spp.) is widely cultivated for the important economic value of its fiber. VW, which caused by *Verticillium dahliae* (*V. dahliae*) [1], is a soil-borne vascular disease and due to year-round continuous cropping and has become the major limiting factor of cotton produce in Xinjiang region. VW is particularly difficult to prevent in cotton and no efficient method has been developed to deal with it. Therefore, breeding and planting VW resistant varieties is the most effective and economical method to control the epidemic. Upland cotton (*Gossypium hirsutum* L.), a tetraploid species that supplies over 97% of the world’s lint fiber (National Cotton Council, http://www.cotton.org, 2006), has been the primary focus of genetic research and breeding. However, due to a scarcity of immune or highly resistant upland cotton germplasm, most commercial cultivars remain susceptible or only tolerant to VW. Consequently, the genetic and molecular mechanisms underlying resistance are poorly understood, and VW-resistant breeding progress of upland cotton has been slow [2].

To analyze the inheritance and enhance upland cotton genetic resistance to VW, researchers have conducted numerous studies to map resistance genes and QTLs using diverse genetic materials, populations under different evaluation environments [3–10]. So far, a lot of VW resistance quantitative trait loci (QTLs) have been reported and distributed on 24 chromosomes except for chromosomes 10 and chromosomes 18 in cotton, and most of these resistant loci were detected in the *G. barbadense* × *G. hirsutum* populations [10]. *G. barbadense* which characterized by extra-long-staple cotton carry high levels of resistance to VW compared to *G. hirsutum* [10–13]. Hence, cotton breeders have made great efforts to introgress VW resistance genetic fragments or gene(s) from *G. barbadense* to *G. hirsutum* and expected to breed new upland cotton cultivars with both high yield and high VW resistance. But most of these resistance fragments or gene(s) have not been successfully transferred into commercial upland cotton due to linkage drag between the resistance and undesired agronomic traits and distortion in segregation of the interspecific hybrid [2, 10]. In China, a large amount of traditional breeding is not a meaningless work for improvement of resistance to VW, and some new germplasm of upland cotton resistant to VW that can withstand production testing has been created and screened through traditional breeding, providing usable germplasm for breeding resistant upland cotton cultivars to VW [14]. Therefore, it will be practicable for locating and cloning the VW resistant sites and genes by utilizing the resistant germplasm of upland cotton as material and achieve the goal of rapid and accurate improvement of upland cotton resistance to VW by molecular marker assisted selection.

With the development of genomic technologies, methods for identifying major loci controlling complex traits have become increasingly efficient. The traditional methods of QTL mapping require detecting the genotype of individual plants to establish linkage groups and collecting phenotype data of individual plants or lines in the population. The bulked segregant analysis (BSA) method in combination with whole-genome resequencing is currently one of the most popular methods of localization of the main effect sites, as it is regarded as an efficient and cost- effective approach to rapidly locate genomic regions, and does not require the construction of linkage mapping [15]. Through high-throughput sequencing technology, genetic differences between extreme phenotype mixed pools are compared, and molecular markers linked with the target trait are quickly screened, significantly improving the accuracy and efficiency of localization. Ren et al. (2019) identified one associated region spanning 5.7 Mb on chromosome 8 for the resistance to Gummy Stem Blight using BSA-seq method [16]. Fang et al. (2025) revealed two stable QTLs for protein and oil content by the joint analysis of QTL mapping and BSA-seq [17]. Gebregziabher et al. (2025) found 16 candidate genomic loci for soybean seed carotenoid contents using next generation sequencing-based BSA [18]. Fu et al. (2024) detected a candidate region of 7.2-Mb on chromosome A09 for the node of the first fruiting branch in upland cotton by BSA-seq, and QTL mapping and t-test based on Kompetitive Allele-Specific PCR (KASP) genotyping and narrowed the candidate region to 260kb [19]. These results showed that the joint analysis of QTL mapping and BSA-seq could identify stable QTL for the complex quantitative traits.

The resolution and accuracy of BSA-seq are influenced by several factors, including the number of extreme-phenotype individuals selected for the bulks, the precision of phenotypic scoring, and the overall population size [20]. Development SNP and InDel markers in BSA-seq localization interval and conducting local maps, QTL mapping analysis can further narrow candidate intervals associated with the target trait on the basis of BSA-seq analysis. In this study, we obtained *G. hirsutum* cv. Jimian 122 (JM122) and Xinluzao 4 (XLZ 4) with significant differences in resistance to VW based on screening and identification in natural disease nursery. Among them, the average disease index (DI) of JM122 at two points in three years is 2.5, belonging to high resistance type, while the average DI of XLZ 4 is 93.2 at two points in three years, with disease incidence of 100%, belonging to high susceptibility type. Using JM122 and XLZ 4 as parents, an F_2_ segregating population consisting of 732 individual plants was constructed. Based on the VW resistance evaluation results under greenhouse conditions of the F_2:3_ family lines, F_2_ individual plants exhibiting extreme resistance and susceptibility to VW were selected to establish two mixed pools. Using the BSA-seq method, four candidate regions for resistance to VW were obtained. KASP molecular markers were developed in the candidate regions to construct local genetic maps based on the genotypes of SNP sites. Using QTL mapping method, two VW resistance QTLs were identified and further confirmed within the four regions detected by the BSA-seq method, which is located on chromosomes A05 and D07, named qVW-A05 and qVW-D07, respectively. The two intervals jointly detected by QTL and BSA-seq mapping methods will be used as the main research targets. Through transcriptome and VIGS functional identification, four candidate genes will be obtained within the two mapping intervals. The result of the study is a typical example of the combination of BSA-seq and QTL mapping for efficiently and accurately location the major loci of quantitative traits. This also conferred the foundation for fine-mapping and cloning of *VW* resistant genes in upland cotton in the later stage.

## Materials and methods

### Plant and fungus materials

*G. hirsutum* cv. JM122 and XLZ 4 showed significant differences in resistance to VW based on screening and identification in natural disease nursery. Among them, *G. hirsutum* cv. JM122, which was introduced from Hebei Academy of Agricultural and Forestry Sciences, the average DI of *G. hirsutum* cv.JM122 is 2.5 at two points for three years. *G. hirsutum* cv. XLZ 4 is bred by the Agricultural Science Institute of the 7th Agricultural Division of the Xinjiang Production and Construction Corps in 1994. The average DI of *G. hirsutum* cv. XLZ 4 is 93.2 at two points for three years, with an disease incidence of 100%, belonging to high susceptibility type. In this study, three *V. dahliae* isolates Bp2, V991 and VD13 were used to identify the VW resistance level of *G. hirsutum* cv. JM122 and XLZ 4 in the glasshouse. Bp2 is a non-defoliating isolate, as representative of middle virulence isolate in Yangtze River cotton growing region. V991 is a prevailing defoliating isolate in the Yellow and the Yangtze River cotton growing regions. VD13 is a defoliating isolate, which was isolated from an infected plant in Xinjiang region by Institute of Plant Protection, Jiangsu Academy of Agricultural Sciences. In 2018, an F_1_ hybrid was generated in Shihezi, Xinjiang, by crossing *G. hirsutum* cv. XLZ 4 (female parent) with *G. hirsutum* cv. JM122 (male parent). In winter of 2018, the F_1_ generation was grown at Hainan Plant Experiment Station and self-pollinated to produce the F_2_ population. In 2019, the F_2_ population containing 732 individuals were planted at Shihezi Plant Experiment Station and self-pollinated to produce the F_2:3_ family lines.

### VW resistance evaluation under greenhouse conditions

The VW resistance evaluation performed under greenhouse conditions was also conducted with two replications from October 2020 to April 2021 using the Chinese national standard method. Sulfuric acid-delinted seeds of each F_2:3_ lines and parents were germinated in paper cups (250 mL) containing a soil mixture of 2:1 vermiculite and peat of volume ratio in a greenhouse. At least 20 plants were inoculated and investigated in per F_2:3_ family lines. The temperature of the greenhouse was controlled approximately 26 °C in the daytime and 22 °C at night. When cotton seedlings grew into two true leaf stage, the paper cup was bottom tore and put into a new paper cup with 30 ml conidial suspensions (3.0 × 10^7^ conidia/ml), and the seedlings were treated under the similar temperature and a high humidity environment. Thirty-five days after inoculation, disease symptoms were scored as described in previous report [4]. The disease grades of 0, 1, 2, 3, and 4 for leaf disease symptoms were as follows: 0: grade indicates healthy, with no disease symptoms; 1: grade indicates ≤ 25.0% leaf exhibited disease symptoms; 2: grade indicates 25.1 to 50.0% leaf exhibited disease symptoms; 3: grade indicates 50.1 to 75.0% leaf exhibited disease symptoms; 4: grade indicates > 75.0% of the leaf surface exhibited disease symptoms, with plants completely defoliated or dead. The VW DI was calculated using the following formula:

$$\mathrm{DI}\left(\%\right)=\frac{\sum_{i=1}^n\mathrm{XiYi}}{4N}\times100$$

X is the disease grade between 0 and 4, Y is the number of plants with corresponding disease grade, and N is the total number of investigated plants of each F_2:3_ family. The Death rate(DR)and the incidence of disease (ID) of the F_2:3_ family were also statistically analyzed, DR= N_4_/*N*×100%, ID= (N_4_+ N_3_)/*N*×100%, N_3_ and N_4_ are the number of plants with grade 3 and grade 4.

*V. dahliae* isolate VD13 used to identify the VW resistance level of F_2:3_ family lines was grown on solid potato sucrose agar culture medium at room temperature (25 °C) for 7 d, then inoculated into liquid potato sucrose culture medium and oscillated for 7 d, and filtrated using 2-layer gauze and diluted to a final concentration of 3 × 10^7^ conidia/ml. All phenotypic data were based on biological replicates of at least 20 individual plants. The statistical significance of differences between groups was assessed using Student’s *t*-test. Differences were considered statistically significant at *P* < 0.05.

### BSA-seq and association analysis of candidate intervals

Based on the identification results of resistance to VW in the greenhouse of the F_2:3_ families after thirty-five days post inoculation, the corresponding F_2_ individual plants were classified into three categories according to the Dl and DR: extreme resistant (SR, the values of Dl and DR are lower than those of the resistant parent, *G. hirsutum* cv.JM122), intermediate (the values of Dl and DR are between those of resistant and susceptible parents), and extreme susceptible (SS, the values of Dl and DR are higher than those of the susceptible parent, *G. hirsutum* cv XLZ 4). The extreme resistant and susceptible bulks were constructed by combining equal amounts of DNA from 30 homozygous resistant F_2_ plants (BR) and 40 homozygous susceptible F_2_ plants (BS), respectively. Although the resistant bulk (30 individuals) and susceptible bulk (40 individuals) differed slightly in sample size, both bulks were subjected to high-depth sequencing (34.51× for the resistant bulk and 41.08× for the susceptible bulk). Such high sequencing depths ensured reliable detection of allele frequency differences even under unequal bulk sizes, thereby minimizing potential bias caused by the discrepancy in sample size. Genomic DNA was extracted from freeze-dried leaf tissue collected from F_2_ seedlings using the CTAB extraction procedure as described by [21]. DNA quality and concentration were determined by agarose gel analysis and spectrophotometry (NanoDrop ND-1000; Thermo Scientific, Wilmington, DE, U.S.A.), and the concentration was adjusted to be in the range of 50 to 100 ng µl^− 1^.

Approximately 2 ug of DNA from the two bulks and the two parental lines was used to construct paired-end sequencing libraries according to the manufacturer’s instructions (Illumina TruSeq library construction). The constructed libraries were subjected to whole-genome resequencing with Illumina HiSeq 2500. Raw sequencing reads were subjected to quality control using FASTQC [22]. Low-quality paired-end reads (Q ≤ 5) with more than 50% bases in any single-end reads or the reads containing adapter (reads containing more than five adapter polluted bases) or poly-N sequences (the number of N bases accounting for > 5% of total bases) were filtered. After removing adapters and low-quality reads, the clean reads were aligned against the reference genome of cotton (TM-1_V2.0, https://www.cottongen.org/species/Gossypium_hirsutum/ZJU-AD1_v2.1) by using Burrows-Wheeler Aligner software [23].The Genome Analysis Toolkit was used to call SNPs and small InDels across the parental lines and bulks with the standard filter method [24]. SNP and InDel index association analysis were used to determine genotype frequency differences between the two bulks by calculating the Δ(SNP index) and Δ(InDel index) [25] In this study, PS represents for the female parent (susceptible, XLZ 4), and PR represents for the male parent (resistant, JM122), aa denotes the genotype from the extreme resistant pool, and bb denotes the extreme susceptible pool. the Δ(SNP index) and Δ(InDel index) values were calculated as follows: SNP-index(bb)= PSbb/(PRbb+PSbb), SNP-index(aa)=PSaa/(PRaa+PSaa), and Δ(SNP-index) = SNP-index(aa)-SNP-index(ab), InDel-index(bb)= PSbb/(PRbb+PSbb), InDel-index(aa)=PSaa/(PRaa+PSaa), and Δ(InDel-index)= InDel-index(aa)།InDel-index(ab), where PSbb indicates the depth of the bb population derived from PS and PRbb indicates the depth of the bb population derived from PR. PSaa indicates the depth of the aa population derived from PS, and PRaa indicates the depth of the aa population derived from PR. A sliding window analysis was applied, averaging the Δ(SNP index) and Δ(InDel index) values within a 1-Mb-sized window and 10-kb step increment using an in-house-developed Python script (available on request) based on the program developed by Takagi et al. (2013) [26]. The average was plotted for all chromosomes and regions in which the averages Δ(SNP index) and Δ(InDel index) of a locus were more close to the target characteristic than that of surrounding regions, and windows that showed an average *P* value < 0.0 L were considered candidate intervals associated with resistance to VW resistance.

### Development and analysis of KASP markers

To further narrow the candidate intervals and verify the BSA-seq results, SNPs were identified between parental lines and the references genome of cotton. The KASP primers for detecting the SNPs carried standard FAM or HEX compatible tails (FAM tail: 5’-GAAGGTGACCAAGTTCATGCT-3’; HEX tail: 5’- GAAGGTCGGAGTCAACGGATT-3’) with the target SNP at the 3’ end. The developed KASP markers were first tested for their ability to differentiate the polymorphism by genotyping the two parents and a few F_2_ individuals with KASP genotyping assays at China Nanjing Genepioneer Biotech Company (Nanjing, China). Assays were tested in 384-well format and set up as 5 µL reactions (1.25µL template, 2.5µL of V4 2× Kaspar mix and 1.25µL primer mix). PCR cycling was performed on a Eppendorf Mastercycler pro 384 using the following protocol: hotstart at 95 °C for 10 min, followed by ten touchdown cycles (95 °C for 20 s; touchdown 61 °C, -0.6 °C per-cycle, 60 s) then followed by 27 cycles of amplification (95 °C 20 s; 55 °C 60 s). The primers that detected clear polymorphism were utilized to genotype the entire mapping population. Genotyping data obtained based on the florescence detected from the KASP assay were viewed and analyzed through KlusterCaller software (LGC Genomics, Beverly, MA, U.S.A.).

### Linkage map construction and QTLs analysis

Linkage map construction and QTL analysis were performed using software QTL IciMapping V3.2 (http://www.isbreeding.net/default.aspx?aspxeorpath=/download software ICIM.aspx) [27]. The linkage map was constructed using the MAP function of the software QTL IciMapping 3.2 with a minimum logarithm of odds (LOD) value of 3.0. Recombination values used to map distances in centimorgans(cM) were calculated based on the Kosambi mapping function [28]. The means of DR, DI and ID of each F_2:3_ family calculated from at least 20 plants per family were used as the phenotypic data for the QTL mapping analysis. The BIP functionality (QTL mapping in biparental populations) was used for QTL analysis in QTL IciMapping. The inclusive composite interval mapping of additive (ICIM-ADD) QTL method with mapping parameters of a walking step of 1.0 centimorgan, and a probability of the stepwise regression of 0.001 was used for significance of QTL identification. Significant LOD threshold value was calculated using 1,000 permutations, with a type I error of 0.05. The confidence interval of the QTL was determined by 1-LOD intervals surrounding the QTL peak, Missing phenotypes were replaced with ‘Deletion’. The values of the additive effect and phenotypic variance of the QTL were also estimated using QTL IciMapping V3.2.

### Virus-induced gene silencing (VIGS) experiment followed by V.D inoculation

Candidate gene fragments were PCR-amplified from JM122 and XLZ 4 cDNA using TRV vector restriction site–specific primers listed in Supplementary Table 1. After digestion with the corresponding restriction enzymes, the products were ligated into the pTRV2:00 vector to construct pTRV2:Target-gene recombinant plasmids. The pTRV2:*GhCLA1* (Cloroplastos alterados 1) causing albino plant was used as positive control. Detailed method was described in previous report Gao et al. (2011) [29]. These vectors and pTRV1 were transformed into *Agrobacterium tumefaciens* strain GV3101 by electroporation. *Agrobacterium* cultures containing pTRV2:Target-gene and pTRV1 were grown overnight at 28 °C in liquid LB medium containing 50 mg/L kanamycin and 25 mg/L rifampicin to OD ≈ 0.8. Cells were centrifuged at 1180 g for 5 min at room temperature; the pellet was collected and resuspended in infiltration medium containing 10 mM MgCl_2_, 10 mM MES and 200 µM acetosyringone. Mix the cell suspensions containing pTRV2:Target gene and pTRV1 at a 1:1 ratio, incubate at room temperature for at least 3 h, and then infiltrate the mixture into the two fully expanded cotyledons of JM122 and XLZ 4 seedlings. The treated seedlings were grown in a controlled environment chamber at 25 °C with 16 h/8 h light/dark photoperiod cycle and relative humidity 80%. After infiltration with pTRV2:*GhCLA1*, and once the leaves displayed an albino phenotype, target-gene expression in all VIGS-treated seedlings was measured by quantitative real-time PCR (qRT-PCR) using specific primers. Subsequently, positive plants with target-gene silencing were inoculated with the *V. dahliae* isolate VD13, following the procedure described above. For each treatment group, 20 plants were scored for disease index, and the experiment was repeated three times. Resistance to Verticillium wilt in each treatment group was assessed strictly according to the evaluation system established earlier in this study. Data are presented as mean ± SD. Student’s *t*-test was used to compare treatment groups with the corresponding empty-vector control (pTRV2:00). Differences were considered statistically significant at *P* < 0.05.

### RNA extraction and expression analysis

Seedlings of JM122 and XLZ 4 display two true leaf stage and the true leave of the pTRV2:*GhCLA1* lines used as a positive control display white-colored after VIGS. The leave of samples was harvested, and immediately frozen in liquid nitrogen for the determination of expression responses later. Total RNA was extracted from cotton root tissues using an E.Z.N.A. PlantRNA Kit (Omega Bio-tek, Norcross, GA, U.S.A.) according to the manufacturer’s instructions. First-strand cDNA was synthesized in a final reaction volume of 20 µl containing 2 µg of RNA according to the instructions of the PrimeScript™ RT reagent Kit with gDNA Eraser Kit (TaKaRa, Japan, Code No. RR047A). The synthesized cDNAs were utilized as templates in the following qRT-PCR reactions. The qRT-PCR was performed on an ABI PRISM 7500 Real-Time PCR System with the SYBR Premix Ex Taq II kit (TaKaRa, Japan, Code No. RR820A) according to the manufacturer’s instructions. The qRT-PCR was carried out with three biological replicates. The cotton Histone 3 gene (Accession number: AF024716) was used as an internal reference. The relative expression level = 2^-△CT^;△CT=(Ct_gene_­Ct_His_). qRT-PCR specific primers were designed with Beacon Designer 7.0 software from Premier Biosoft International, Palo Alto, CA.

### Transcriptome analysis

The root tissues of *G. hirsutum* cv. JM122 and XLZ 4 were harvested 0, 24 h, 48 h after inoculation with *V. dahliae* isolate VD13 and water as control. The total RNA was extracted and assessed using an Agilent 2100 Bioanalyzer system (Agilent Technologies, Palo Alto, CA, U.S.A.) and determined by RNase free agarose gel electrophoresis. After extraction of the total RNA, the mRNA was enriched by Oligo(dT) beads. Sequencing was subsequently performed using an Illumina HiSeq2500 system by Gene Denovo Biotechnology Co. (Guangzhou, China). The reads were further filtered using fastp (version 0.18.0) to obtain high quality clean reads. The fragment per kilobase of transcript per million mapped reads (FPKM) value was calculated for each transcript for quantifying the expression abundance and variations using the StringTie software. The samples under *V. dahliae* and water treatment were used as *V. dahilae* inoculation and mock treatment in this study. The differential RNA expression between the two groups was analyzed using theDESeq2 software. Genes with a false discovery rate (FDR) < 0.05 and absolute fold change (FC) ≥ 2 were regarded as the differentially expressed genes (DEGs). Three plants at each treated period were mixed as a biological replicate. In this study, three independent biological replicates were set up.

## Results

### VW resistance evaluation of the parents and the F_2:3_ population in greenhouse investigations

**Fig. 1:**
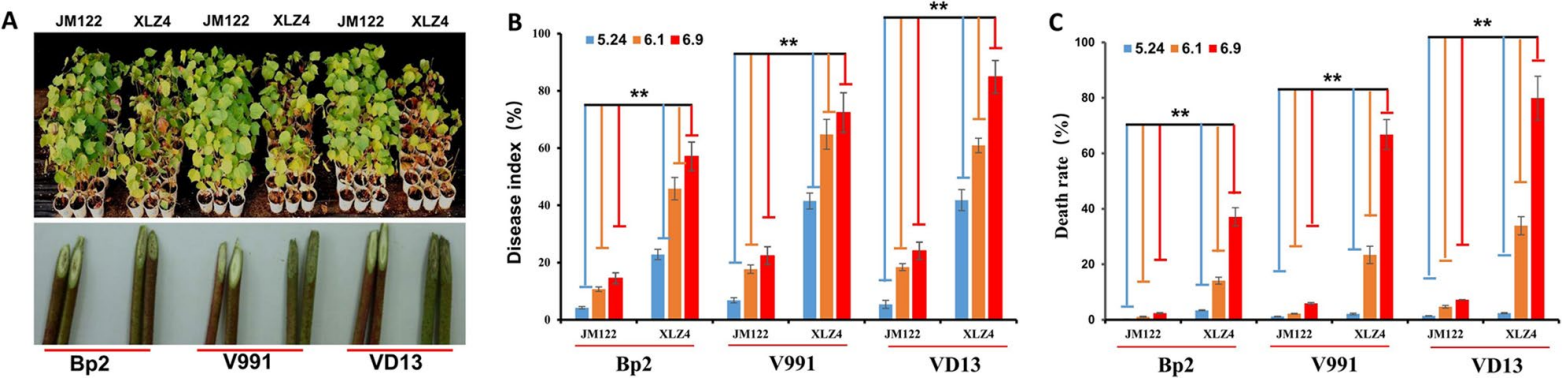
The resistance phenotype of two parents, JM122 and XLZ 4 in artificial inoculation three *V. dahliae* isolates Bp2, V991 and VD13 in 2017 in Nanjing. **A**: The resistance phenotype and vascular bundle discoloration of JM122 and XLZ 4. The picture was taken 5 weeks after inoculation. **B**: The DI of two parents in artificial inoculation three *V. dahliae* isolates Bp2, V991 and VD13. **C**: The DR of two parents in artificial inoculation three *V. dahliae* isolates Bp2, V991 and VD13. The results were first evaluated at 28d after V. dahliae inoculation from three replications with at least 20 plants. 5.24, 6.1 and 6.9 (May 24th, June 1st and June 9th) represent the time of three investigations at intervals of 8 days, respectively. The data are derived from three independent biological replicates, and the error bars represent the standard deviation (SD). Asterisks indicate statistically significant difference, as determined by the Student’s *t*-test (**P < 0.01)

In April 2019, the resistance level of parental lines to three *V. dahliae* isolates Bp2, V991 and VD13 were evaluated in the glasshouse. The results of resistance identification showed that the resistance of *G. hirsutum* cv. JM122 and XLZ 4 to all three *V. dahliae* isolates appear significant differences. Among them, *G. hirsutum* cv. XLZ 4 is extremely sensitive to the isolates VD13 and V991 and the DI can reach over 40 after 28 days post inoculation. After 42 days post inoculation, *G. hirsutum* cv. XLZ 4 inoculated with the isolate VD13, compared to *V. dahliae* isolates Bp2 and V991, showed the highest DR and DI, reaching 79.9% and 84.9% (Fig. 1). These results show that there is a significant difference in the resistance of JM122 and XLZ 4 to VW. In contrast to isolates Bp2 and V991, inoculation with VD13 resulted in more consistent disease phenotypes across replicates for both parents.

**Fig. 2:**
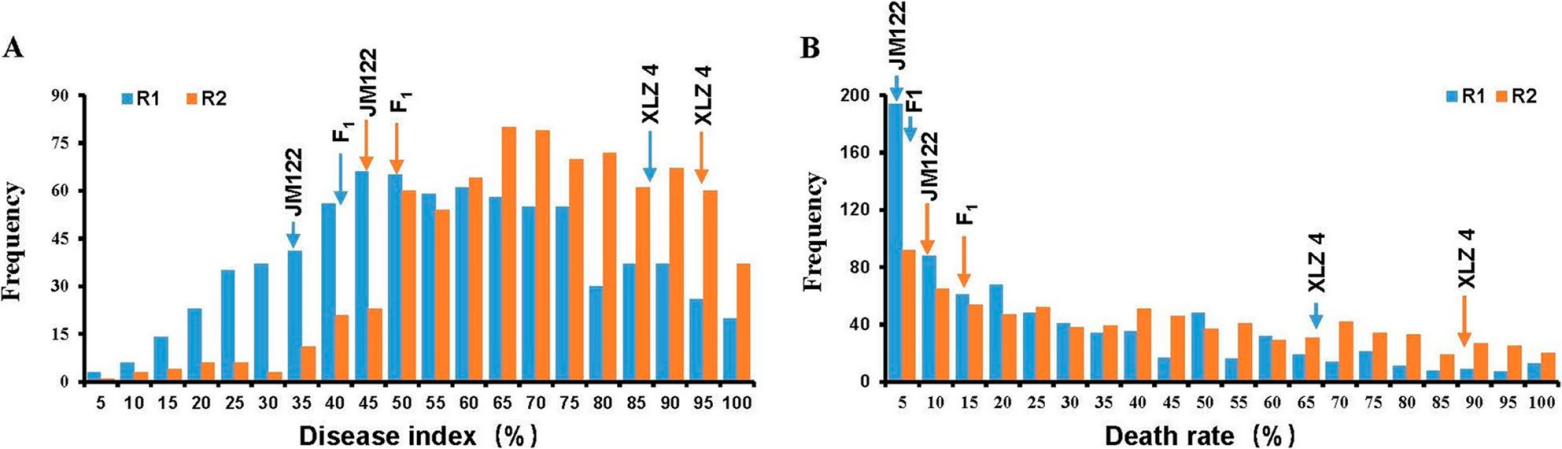
Frequency distributions of disease index and Death rate for two independent repeated VW resistance evaluation experiments in greenhouse. **A**: Frequency distributions of disease index. **B**: Frequency distributions of Death rate. R1 and R2 denote two VW resistance evaluation experiments that were performed from October 2020 to April 2021 using the Chinese national standard method in the same greenhouses, respectively

The evaluations of VW resistance for 732 F_2:3_ family lines were performed two times in the greenhouse using *V. dahliae* isolate VD13, and the frequency distributions of different resistance levels of the parents and F_2:3_ family lines are shown in Fig. 2; Table 1. The DI values of the F_2:3_ population varied from 8.33% to 100% in first time experiment, from 23.33% to 100% in second time experiment. The DI values of the F_1_ population were 38.12% and 51.86%, which was close to that of the resistant parent JM122 (31.84% and 44.29%), and the mean DI values were 55.15% and 70.20% for the first- and second-time experiments, respectively. The DR values of the F_2:3_ population varied from 0 to 100% in the first- and second-time experiments. The DR values of the F_1_ population were 3.29% and 13.84%, which was also close to that of the resistant parent JM122 (2.87% and 6.6%), and the mean DR values were 26.98% and 43.38% for the first- and second-time experiments, respectively. The above results indicate that JM122 has high and broad-spectrum resistance to VW and the resistance level of F_2:3_ population has abundant variation making it suitable for mapping of VW resistance sites.

**Table 1:** Descriptive statistics and broad-sense heritability (H_2_) of disease index in greenhouse tests

Traits	Test	F_2_ population						Parent			F_1_	H_2_
		Mean	Min	Max	SD	Skewness	Kurtosis	JM122	XLZ 4	Midparent		
DI	R1	55.15	8.33	100.00	17.82	-0.207	-0.501	31.84	85.26	58.55	38.12	0.74
	R2	70.20	23.33	100.00	14.44	-1.032	1.587	44.29	94.29	69.29	51.86	
DR	R1	26.98	0.00	100.00	21.05	0.921	-0.069	2.87	67.86	35.37	3.29	0.68
	R2	43.38	0.00	100.00	23.97	0.230	-1.052	6.60	87.86	47.23	13.84	

DI and DR represent disease index and Death rate, respectively. R1 and R2 represent two independent repeated VW resistance evaluation experiments which were performed from October 2020 to April 2021 in the same greenhouse, respectively. *SD* Standard deviation

### BSA-seq data analysis and identification of candidate intervals

30 homozygous resistant and 40 homozygous susceptible F_2_ plants were used to form two bulks for whole-genome resequencing. In total, 314.47 Gb Clean data of high quality (93.07% ≥ Q30 ≥ 92.87%) were obtained by filtering and trimming from 314.77 Gb Raw data generated through parental and bulks genome resequencing. The GC content ranged from 35.00% to 35.31%. the clean data from four samples were aligned to the cotton reference genome sequence, and the average sequence depths were 15.36-fold for resistant parent JM122, 15.24-fold for susceptible parent XLZ 4, 34.51-fold for extreme resistant bulk (BR), 41.08-fold for extreme susceptible bulk (BS). The number of mapped reads was 233,733,966 (98.49%) in RP (JM122); 229,001,104 (99.48%) in SP (XLZ 4); 523,475,478 (99.23%) in BR: and 623,612,958 (99.20%) in BS (Table 2). The above results indicate that the quality of resequencing data is reliable and has a high level of mapping to the reference genome, which can be used for subsequent mutation detection and related analysis.

**Table 2:** Summary of sequencing data and the data aligned to the cotton reference genome for the parental lines and the resistant and susceptible pools

Samples	Clean reads	Clean data	GC content (%)	Q30 (%)	Ave_depth	No. of Read	Mapped reads (%)	No. of SNPs
RP(JM122)	116,866,983	35,060,094,900	35.00	92.87	15.36	233,733,966	98.49	1,878,106
SP(XLZ 4)	114,500,552	34,350,165,600	35.25	92.87	15.24	229,001,104	99.48	2,449,490
BR	261,737,739	78,521,321,700	35.31	92.93	34.51	523,475,478	99.23	2,896,427
BS	311,806,479	93,541,943,700	35.27	93.07	41.08	623,612,958	99.20	2,904,209

*RP * resistant parent, JM122, *SP* susceptible parent, XLZ 4, *BR * resistance bulk, and *BS * susceptible bulk

Clean reads: Total number of pair-end reads in the clean data

Clean data: Total number of bases in the clean data

Ave_depth: Average read depth of each sample

No. of Read: Number of reads aligned to the cotton reference genome. Both ends are counted separately

Mapped reads: Percentage of reads aligned to the cotton reference genome

No. of SNPs: Number of Single Nucleotide Polymorphisms

**Fig. 3:**
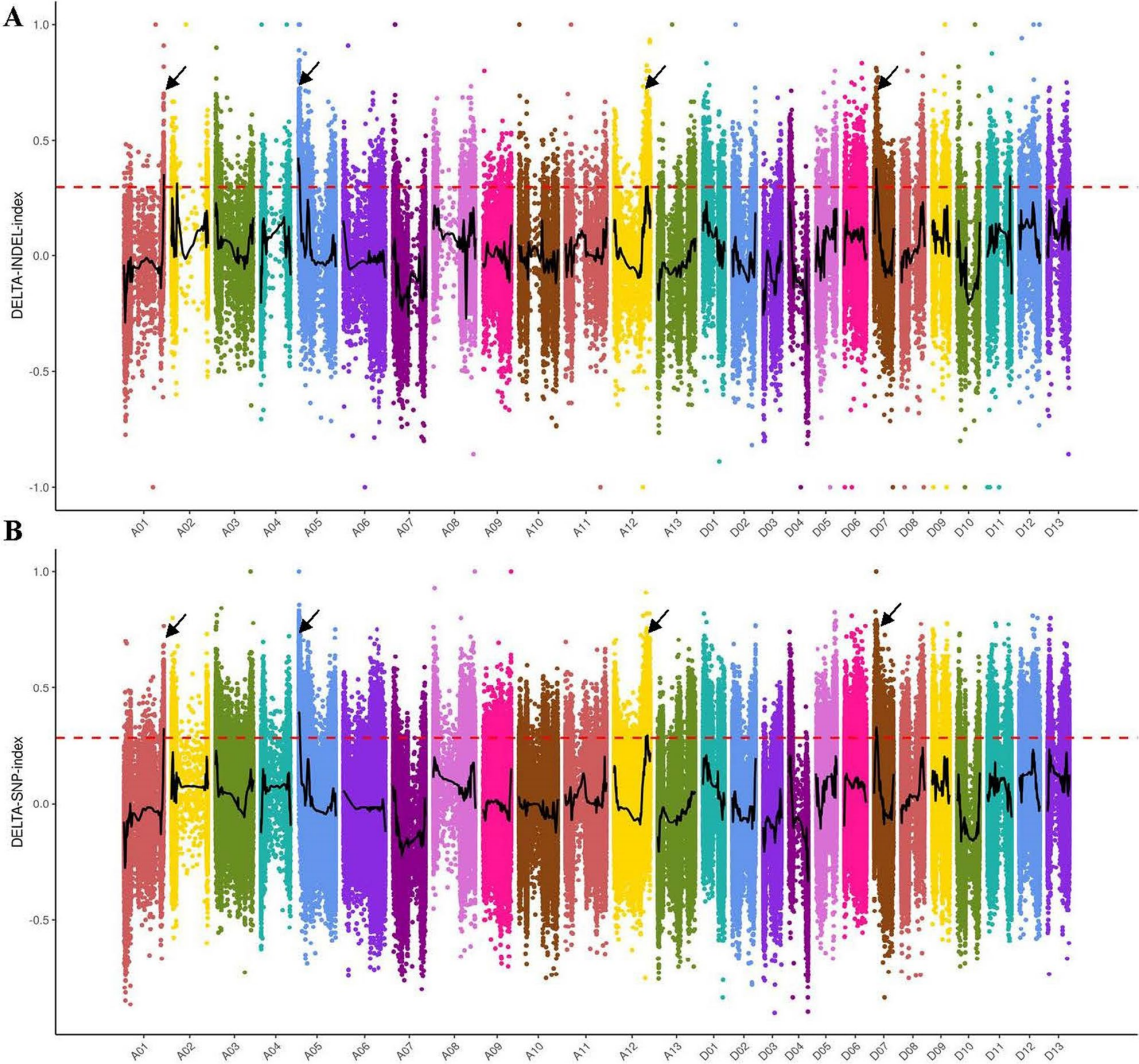
The results of association analysis using SNP and InDel markers. **A** and **B** represent the results of bulked segregant analysis by calculating the Δ(InDel-index) and Δ(SNP-index) in combination with next-generation sequencing, respectively. The black arrow represents the identified regions of the resistance to VW. The x-axis represents chromosomal position, and the y-axis represents Δ(InDel-index) and Δ(SNP-index) values. The black lines are average Δ(InDel-index) and Δ(SNP-index) values determined by sliding window analysis. The dashed line indicates the association threshold values at the significance level of *P* < 0.01, which was calculated by Loess regression

A total of 1,878,106, 2,449,490, 2,896,427, and 2,904,209 SNPs were identified in RP, SP, BR, and BS, respectively, by comparison with the reference genome (Table 2), and 166,666 (RP_VS_SP), 17,869 (BR_VS_BS), 152,463 (RP_VS_BR), 166,718 (RP_VS_BS), 151,037 (BR_VS_SP) and 157,628 (BS_VS_SP) InDels were obtained (Supplementary Table 2). With the criteria for selecting the SNPs and InDels that are homozygous for each parent and show polymorphism between the parents, in total, 1,769,387 and 17,658 high-quality SNPs and InDels that are polymorphic between the extreme resistant and the susceptible bulks were used for subsequent SNP-index, InDel-index, ∆(SNP-index) and ∆( InDel-index) calculation. The identification of resistance-related candidate regions was accomplished by screening genotypic frequency differences between the extreme resistant and the susceptible bulks. Four regions on chromosomes A01, A05, A12 and D07 with average Δ(SNP-index) and Δ(InDel-index) values above the confidence value (99% [red line]) (Fig. 3) were defined. The candidate genes are anchored to specific intervals of chromosomes A01, A05, A12 and D07. On chromosome A01, the region extends from 117,625,142 to 118,070,610 bp, encompassing a total of 53 genes; chromosome A05 includes a region that spans from 550,472 to 5,126,569 bp, which houses 518 genes; chromosome A12 contains a region from 93,876,666 to 94,556,220 bp, comprising 27 genes; chromosome D07 features a region that spans from 5,637,004 to 9,800,513 bp, with a total of 310 genes identified through SNP association analysis. For the InDel analysis results, the same candidate intervals were detected on chromosomes A01, A05, A12 and D07. However, compared with the SNP analysis results, the candidate interval of the InDel analysis has been narrowed, except for the candidate interval on chromosome A05. Specifically, the candidate intervals on chromosome A01 span from 117,802,585 to 118,037,277 bp and encompass 33 genes; chromosome A05 extend from 356,280 to 5,072,460 bp, containing 523 genes; chromosome A12 are flanked by 94,245,077 and 94,432,668 bp, comprising 7 genes. Lastly, chromosome D07 range from 5,958,289 to 8,807,473 bp, containing 224 genes (Table 3). Integrating SNP and InDel association analysis results, the associated regions were identified on chromosomes A01, A05, A12 and D07, and spanned 0.22 Mb (117,802,585 to 118,037,277), 4.31 Mb (550,472 to 5,072,460), 0.19 Mb (94,245,077 to 94,432,668), and 2.85 Mb (5,958,289 to 8,807,473), respectively.

**Table 3:** The statistical results of associated regions using SNP and InDel by the BSA

Types of markers	Chr.	Start position	End position	Size(Mb)	Genes
SNP associated regions	A01	117,625,142	118,070,610	0.45	53
	A05	550,472	5,126,569	4.58	518
	A12	93,876,666	94,556,220	0.68	27
	D07	5,637,004	9,800,513	4.16	310
InDel associated regions	A01	117,802,585	118,037,277	0.22	33
	A05	356,280	5,072,460	4.72	523
	A12	94,245,077	94,432,668	0.19	7
	D07	5,958,289	8,807,473	2.85	224

### Narrowing and validating the candidate intervals using QTL mapping

**Fig. 4:**
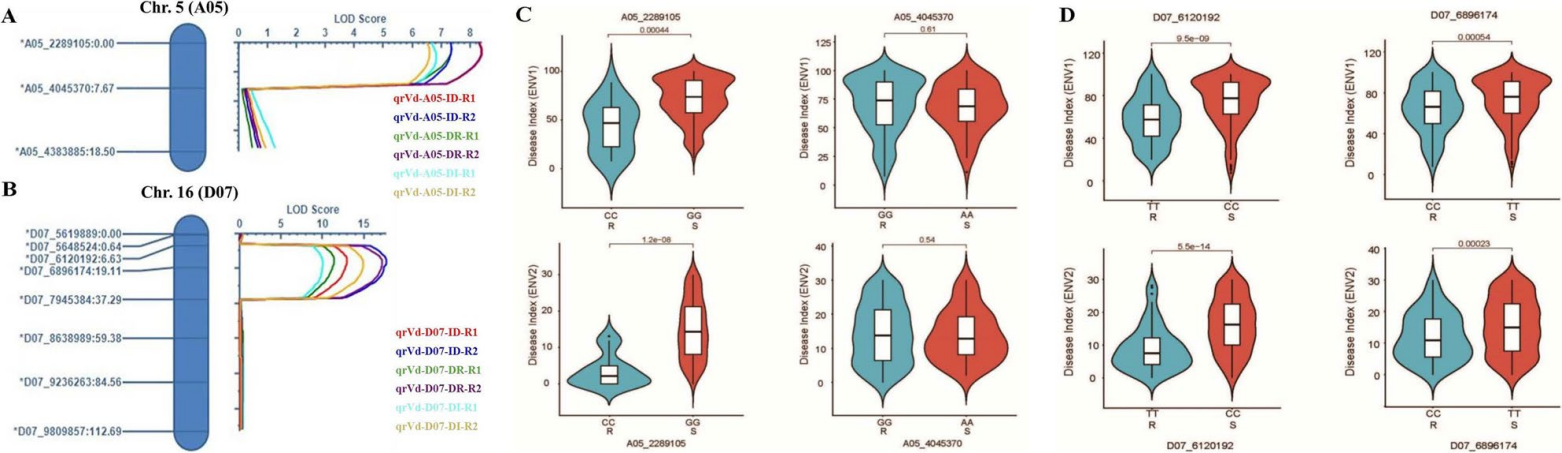
The QTL mapping of VW resistance on the identified regions and the association analysis of the linkage markers. **A** and **B** represent the results of the QTL mapping the major resistance QTL for VW in Chr. 5(A05) and Chr.16(D07), respectively. The physical locations and map distances (in centimorgans) were showed on the left side of Chromosome. The representation format is Chromosome name_physical locations: map distances. The different lines represent the different traits about the resistance to VW. DI, DR and ID were the abbreviations of disease index, death rate and incidence of disease. R1 and R2 represent two VW resistance evaluation experiments in the same greenhouse, respectively. **C** and **D** represent the results of the association analysis of the linkage markers with the qVW-A05 and qVW-D07 in two different environments. The x-axis represents the disease indexes, and the y-axis represents the genotypes of linkage markers. ENV1 and ENV2 represent two different environments. Horizontal dashed lines indicate the LOD significance threshold (*P* < 0.05) determined by 1,000 permutation tests

To further narrow and validate the associated regions, in total, 23 KASP markers were designed from the corresponding sequence of the region harboring SNPs surrounding the resistance loci and tested for polymorphisms with the two parental lines and 50 individuals of the population (Supplementary Table 3). Using KASP assays, genotyping results showed that 17 KASP markers present the biallelic codominant polymorphism expected between the resistant and susceptible parents and in the F_2_ population (Supplementary Fig. 1). The QTL analysis showed that two major QTLs, designated as qVW-A05 and qVW-D07, were detected in both repeats using the phenotypic value of DI, DR and ID, which were flanked by the KASP markers A05_2289105 and A05_4045370, D07_6120192 and D07_6896174, respectively (Fig. 4A, B).

The QTL qVW-A05 explained 8.72% and 8.39% phenotypic variations with LOD scores ranging from 7.4 to 8.4 using the phenotypic value of ID, and explained 7.95% and 8.79% phenotypic variations with LOD scores ranging from 7.3 to 8.4 using the phenotypic value of DR, and explained 8.80% and 8.61% phenotypic variations with LOD scores ranging from 6.6 to 6.8 using the phenotypic value of DI, respectively. The QTL qVW-D07 explained 10.8% and 13.6% phenotypic variations with LOD scores ranging from 12.9 to 17.7 using the phenotypic value of ID, and explained 11.5% and 13.3% phenotypic variations with LOD scores ranging from 11.4 to 17.2 using the phenotypic value of DR, and explained 12.5% and 12.0% phenotypic variations with LOD scores ranging from 10.0 to 14.9 using the phenotypic value of DI, respectively (Table 4). Taken together, the results of QTL mapping validated that there are two major VW resistance loci for *V. dahliae* isolate VD13 on the chromosome A05 and D07, and narrowed successfully the associated regions from 4.31 Mb (550,472 to 5,072,460) to 1.67 Mb (2,289,105 to 4,045,370) and contained 195 genes on the chromosome A05 and from 2.85 Mb (5,958,289 to 8,807,473) to 0.74 Mb (6,120,192 to 6,896,174) and contained 68 genes on the chromosome D07 (Supplementary Table 4). In summary, the QTL mapping results validated both BSA-detected intervals and narrowed them to 1.67 Mb on A05 and 0.74 Mb on D07. These refined intervals are fully contained within the original BSA-seq regions.

**Table 4:** QTL analysis for VW resistance detected using the phenotypic values of ID, DR and DI in the F_2:3_ generations

QTL Name		Chr.	Flanking Markers	LOD values	PVE(%)	Additive	Dominance
qVW-A05	qrVd-A05-ID-R1	A05	A05_2289105-A05_4045370	8.4	8.72	0.10	0
	qrVd-A05-ID-R2	A05	A05_2289105-A05_4045370	7.4	8.39	0.09	-0.01
	qrVd-A05-DR-R1	A05	A05_2289105-A05_4045370	7.3	7.95	0.08	0
	qrVd-A05-DR-R2	A05	A05_2289105-A05_4045370	8.4	8.79	0.10	0
	qrVd-A05-DI-R1	A05	A05_2289105-A05_4045370	6.8	8.80	0.07	0.01
	qrVd-A05-DI-R2	A05	A05_2289105-A05_4045370	6.6	8.61	0.05	0
qVW-D07	qrVd-D07-ID-R1	D07	D07_6120192-D07_6896174	12.9	10.8	0.12	0
	qrVd-D07-ID-R2	D07	D07_6120192-D07_6896174	17.7	13.6	0.13	0.01
	qrVd-D07-DR-R1	D07	D07_6120192-D07_6896174	11.4	11.5	0.10	0
	qrVd-D07-DR-R2	D07	D07_6120192-D07_6896174	17.2	13.3	0.13	0
	qrVd-D07-DI-R1	D07	D07_6120192-D07_6896174	10.0	12.5	0.08	0.01
	qrVd-D07-DI-R2	D07	D07_6120192-D07_6896174	14.9	12.0	0.08	0

DI, DR and ID represent disease index, Death rate and incidence of disease, respectively. R1 and R2 represent two independent repeated VW resistance evaluation experiments which were performed from October 2020 to April 2021 in the same greenhouse, respectively. *PVE:* Phenotypic variation explanation

To further assess the utility and robustness of qVW-A05 and qVW-D07, we performed an association analysis using four markers linked to the two QTLs in a natural population of 468 upland cotton accessions. Phenotypic evaluation of VW resistance was conducted under natural infection conditions in a disease nursery for two consecutive years, and the marker-trait associations were analyzed based on genome resequencing data. The results showed that three of the four markers are significantly correlated with upland cotton VW resistance, and the VW resistance level of cotton varieties with the same genotype as the VW resistant parent JM122 was significantly higher than cotton varieties with the same genotype as the VW susceptible parent XLZ 4. In natural populations, the marker A05_4045370 did not show association with VW resistance, which may indicate that QTL located on chromosome A05 is closer to the marker A05_2289105 (Fig. 4C, D). These results indicated that three linkage markers with qVW-A05 and qVW-D07 can be used for improvement cotton VW resistance by molecular marker assisted selection.

### Analysis of expression profiles

**Fig. 5:**
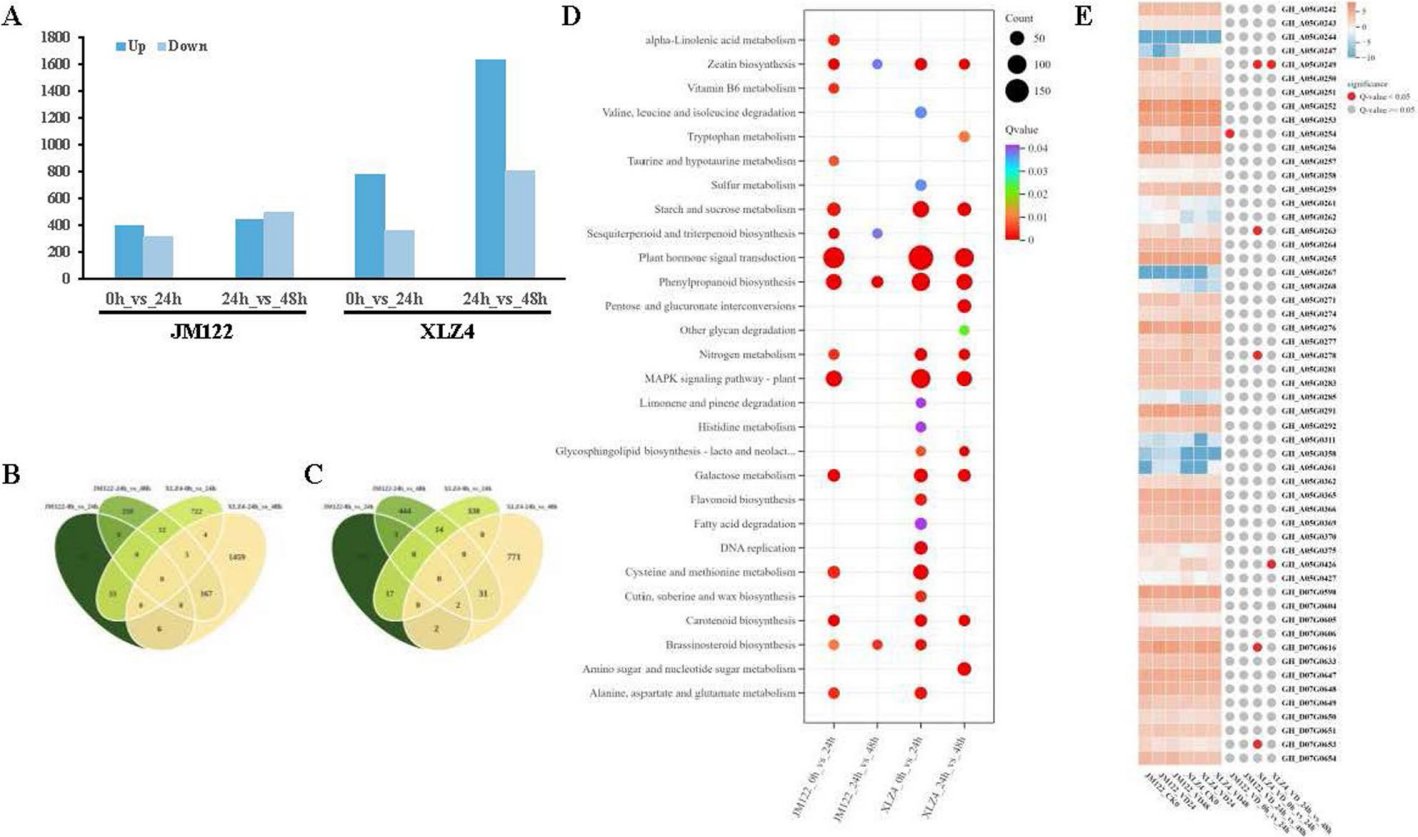
Analysis of differently expressed genes (DEGs). **A**: The number of up-regulated and down-regulated DEGs in four comparison groups; **B-C**. Venn diagram of up-regulated and down-regulated DEGs in four comparison groups; **D**. KEGG enrichment analysis of DEGs; **E**. Heat map diagram of mutated genes in QTL intervals. Differential expression gene analysis was performed using the DESeq2 software, with a significance threshold of FDR < 0.05 and |FC| ≥ 2

Through comparative analysis of differentially expressed genes (DEGs) in the transcriptomes of parent lines JM122 and XLZ 4 at 0 h, 24 h, and 48 h post-inoculation with *V. dahliae* (comparison groups: 0h_vs._24h and 24h_vs._48h), it was observed that the resistant parent JM122 exhibited a total of 710 DEGs (396 up-regulated and 314 down-regulated) during the 0h_vs._24h group. In the 24h_vs._48h group, a total of 935 DEGs were identified (441 up-regulated and 494 down-regulated). In contrast, the susceptible parent XLZ 4 displayed a greater number of DEGs across the corresponding time intervals, and 1,135 DEGs (774 up-regulated and 361 down-regulated) were detected during the 0h_vs._24h group, while 2,445 DEGs (1,639 up-regulated and 806 down-regulated) were identified in the 24h_vs._48h group (Fig. 5A).

Notably, no DEGs were shared between the two parents across the two treatment intervals. In the resistant parent JM122, no up-regulated genes were identified between the 0 h vs. 24 h and 24 h vs. 48 h comparisons. In contrast, the susceptible parent XLZ 4 exhibited seven up-regulated genes (*GH_A05G0333*, *GH_A05G3808*, *GH_A09G2276*, *GH_A10G0459*, *GH_D01G1611*, *GH_D09G2211*, and *GH_D13G2413*). For down-regulated genes, the resistant cultivar JM122 showed five DEGs (*GH_A01G0291*, *GH_A13G1370*, *GH_D04G0278*, *GH_D05G1205*, and *GH_D13G1919*), whereas no down-regulated genes were detected in XLZ 4 for the same comparisons (Fig. 5B, C).

KEGG enrichment analysis of DEGs from both resistant and susceptible parent indicated that the number of metabolic pathways enriched in the susceptible parent was substantially higher than that in the resistant parent. The pathways with the maximum number of enriched genes were Plant hormone signal transduction, MAPK signaling pathway, and Phenylpropanoid biosynthesis. In the resistant parent, only the Phenylpropanoid biosynthesis pathway was significantly enriched across both time intervals. In contrast, all three pathways were enriched in the susceptible cultivar, albeit with a reduced number of involved genes in the later stage (Fig. 5D).

To further screen candidate genes, we integrated the results of QTL mapping, BSA mapping and transcriptome data. Through variant analysis of BSA-seq data, genes harboring mutations classified as missense variants, codon deletions, frameshift mutations, splice-donor site mutations, codon changes plus codon deletions, and codon insertions were selected. A total of variant 55 genes were identified within QTL intervals. Among these, 42 variant genes were located on chromosome A05 (QTL interval: 2,289,105 to 4,045,370), and 13 were located on chromosome D07 (QTL interval: 6,120,192 to 6,896,174). Among these variant genes, seven genes (*GH_A05G0249*, *GH_A05G0254*, *GH_A05G0263*, *GH_A05G0278*, *GH_A05G0426*, *GH_D07G0616* and *GH_D07G0653*) showed significant differential expression in the transcriptomic analysis (Fig. 5E).

Based on the above analyses, we established the following candidate gene screening criteria for selecting genes for VIGS validation: (1) located within the confidence intervals of qVW-A05 or qVW-D07; (2) showing differential expression (|FC| ≥ 2, FDR < 0.05) in the transcriptome data at least at one time point after pathogen inoculation; and (3) possessing nonsynonymous mutations or Indels between the parental lines that may affect protein function. According to these criteria, we selected 16 candidate genes from the A05 interval and 9 candidate genes from the D07 interval (Supplementary Table 5) for subsequent functional validation via VIGS.

### Screening of candidate genes by virus-induced gene silencing (VIGS)

**Fig. 6:**
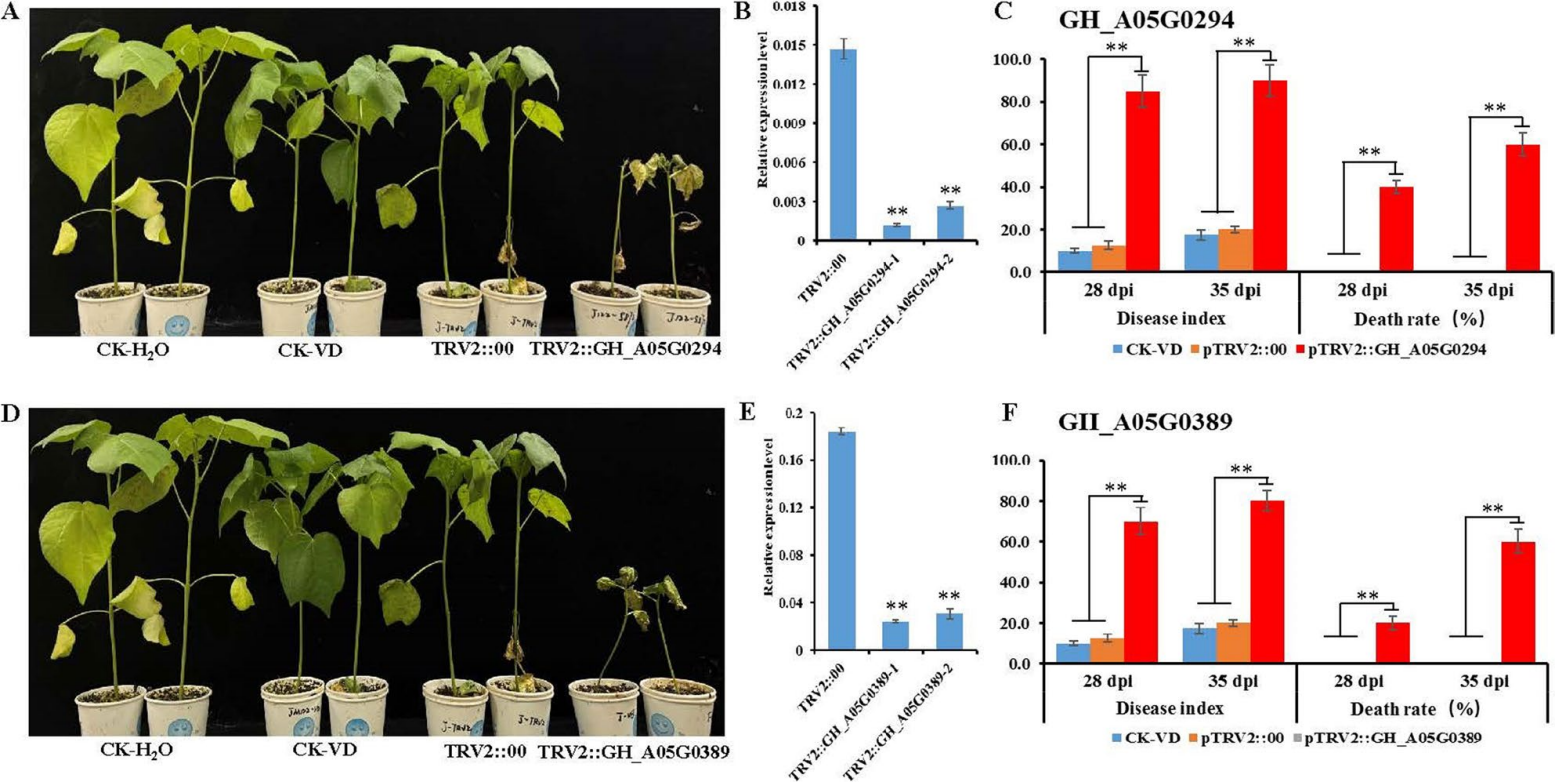
The resistance function analysis of candidate genes in Chr.A05 intervals by VIGS. **A** and **D**: The phenotypes of JM122 under infection by *V. dahliae* isolate VD13 after VIGS with *Agrobacterium* carrying pTRV2:*GH_A05G0294*, pTRV2:*GH_A05G0389* and pTRV2:00. **B** and **E**: qRT-PCR analysis the expression levels of *GH_A05G0294* and *GH_A05G0389* genes in the silenced lines of JM122. **C** and **F**: The DI and DR of JM122 with the silenced *GH_A05G0294* and *GH_A05G0389* gene. The DI and DR of plants were evaluated at 28d and 35d after *V. dahliae* inoculation from three replications with at least 20 plants. CK-H2O and CK-VD represent the phenotype of mock inoculation plants with water and infection by V. dahliae, respectively. Photos were taken at 35 d after *V. dahliae* inoculation. dpi is short for day post inoculation. The data are derived from three independent biological replicates, and the error bars represent the standard deviation (SD). Asterisks indicate statistically significant differences, as determined by the Student’s *t*-test (**P < 0.01)

**Fig. 7:**
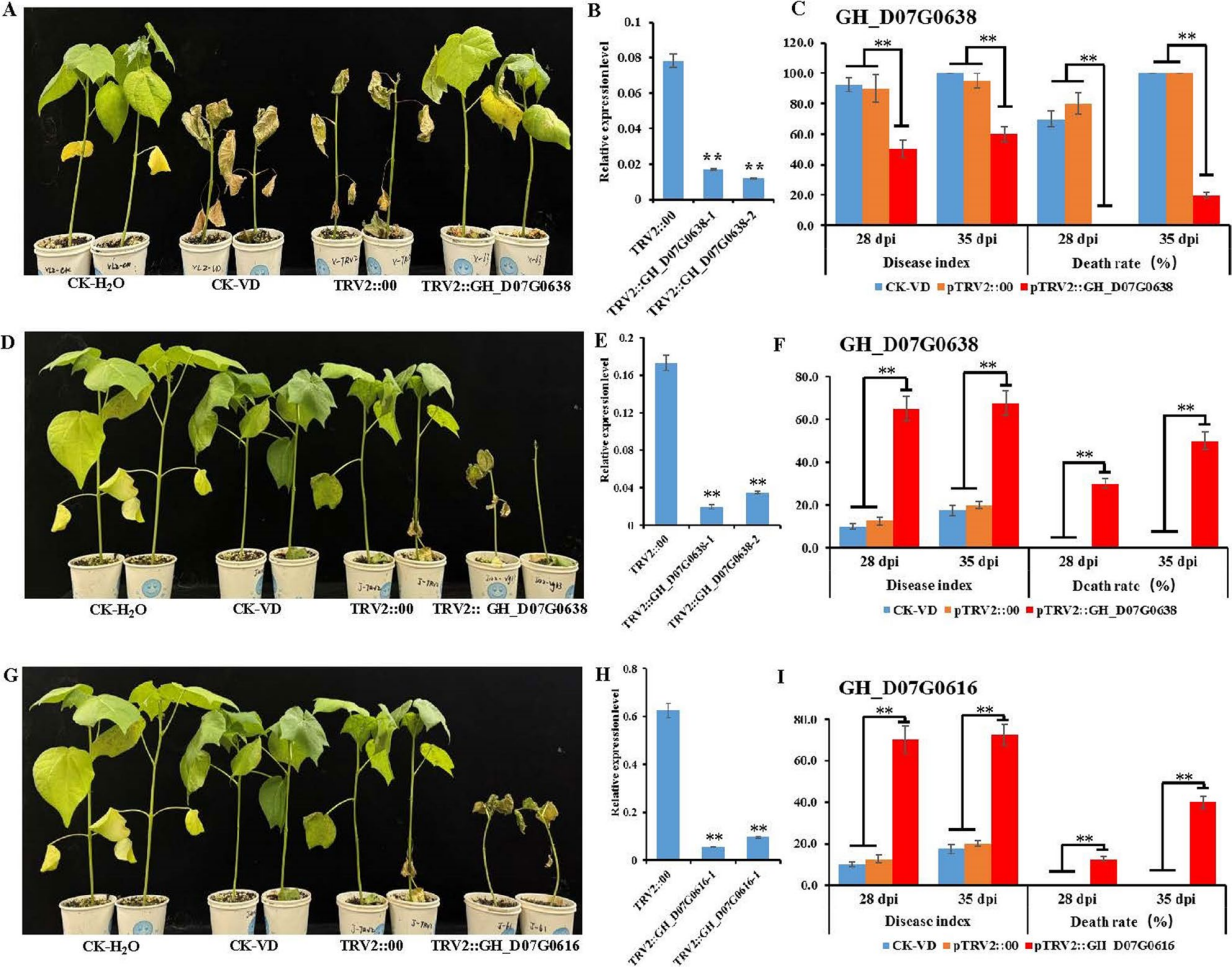
The resistance function analysis of candidate genes in Chr.D07 intervals by VIGS. **A**: The phenotypes of XLZ 4 under infection by *V. dahliae* isolate VD13 after VIGS with Agrobacterium carrying pTRV2:*GH_D07G0638* and pTRV2:00. **B**: qRT-PCR analysis the expression levels of *GH_D07G0638* genes in the silenced lines of XLZ 4. **C**: The DI and DR of XLZ 4 with the silenced *GH_D07G0638* gene. **D** and **G**: The phenotypes of JM122 under infection by *V. dahliae* isolate VD13 after VIGS with *Agrobacterium* carrying pTRV2: *GH_D07G0638*, pTRV2:*GH_D07G0616* and pTRV2:00. **E** and **H**: qRT-PCR analysis the expression levels of *GH_D07G0638* and *GH_D07G0616* genes in the silenced lines of JM122. **F** and **I**: The DI and DR of JM122 with the silenced *GH_D07G0638* and *GH_D07G0616* gene. The DI and DR of plants were evaluated at 28d and 35d after *V. dahliae* inoculation from three replications with at least 20 plants. CK-H2O and CK-VD represent the phenotype of mock inoculation plants with water and infection by V. dahliae, respectively. Photos were taken at 35 d after *V. dahliae* inoculation. dpi is short for day post inoculation. The data are derived from three independent biological replicates, and the error bars represent the standard deviation (SD). Asterisks indicate statistically significant differences, as determined by the Student’s *t*-test (**P < 0.01)

To further reduce the range of candidate genes in QTL intervals, VIGS was applied to analyze the roles of 25 differentially expressed genes resistance to VW based on the results of expression and sequence analysis. *V. dahliae* isolate VD13 was used to inoculate for evaluation the VW resistance of deficient lines compared to the control pTRV2:00 line. The cotton *GhCLA1* gene was used as a positive control with a phenotype of white-colored leave after VIGS in cotton (Supplementary Fig. 2). The results of qRT-PCR analysis showed that the expression levels of 25 genes in deficient lines decreased significantly in seedlings 2 weeks after VIGS infiltration compared to the control pTRV2:00 line (Figs. 6B and E and 7B, E and H). The DI and DR were calculated at 28d and 35d after inoculation. According to the statistical results of the DI and DR, the deficient lines of *GH_A05G0294*, *GH_A05G0389*, *GH_D07G0638* and *GH_D07G0616* genes can significantly impact on the resistance of JM122 and XLZ 4 to VW (Figs. 6A and D and 7A, D and G). For gene *GH_A05G0294*, the DI and DR of JM122 deficient lines reached to 85.2 (DI, 28 dpi), 90.8 (DI, 35 dpi) and 41.2% (DR, 28 dpi), 59.8% (DR, 35 dpi) after VIGS infiltration compared to 10.0 (DI, 28 dpi, JM122, control plant), 12.5 (DI, 35 dpi, JM122, pTRV2:00) and 0 (DR, 28 dpi, JM122, control plant), 0.4% (DR, 35 dpi, JM122, pTRV2:00), respectively (Fig. 6C). For gene *GH_A05G0389*, the DI and DR of JM122 deficient lines reached to 69.8 (DI, 28 dpi), 80.5 (DI, 35 dpi) and 19.5% (DR, 28 dpi), 60.4% (DR, 35 dpi) after VIGS infiltration (Fig. 6F). For gene *GH_D07G0616*, the DI and DR of JM122 deficient lines reached to 70.0 (DI, 28 dpi), 72.5 (DI, 35 dpi) and 12,5% (DR, 28 dpi), 40.0% (DR, 35 dpi) after VIGS infiltration (Fig. 7I). A very interesting result was shown for *GH_D07G0638* gene, the DI and DR of JM122 deficient lines reached to 65.0 (DI, 28 dpi), 67.5 (DI, 35 dpi) and 30.0% (DR, 28 dpi), 50.0% (DR, 35 dpi) after VIGS infiltration (Fig. 7F). However, for susceptible parent XLZ 4, the VW resistance level of the deficient lines of *GH_D07G0638* gene improved after VIGS infiltration, and the DI and DR reached to 50.0 (DI, 28 dpi), 60.5 (DI, 35 dpi) and 0 (DR, 28 dpi), 20.0% (DR, 35 dpi) compared to 92.5 (DI, 28 dpi, XLZ 4, control plant), 100.0 (DI, 35 dpi, XLZ 4, pTRV2:00) and 70.0% (DR, 28 dpi, XLZ 4, control plant), 100.0% (DR, 35 dpi, XLZ 4, pTRV2:00), respectively (Fig. 7C). These results indicate that *GH_A05G0294*,* GH_A05G0389*,* GH_D07G0638* and *GH_D07G0616* screened in this study play important roles in resistance to VW and were as candidate genes in qVW-A05 and qVW-D07 regions. A very interesting result was observed for *GH_D07G0638*. Silencing of this gene in the resistant parent JM122 significantly compromised resistance, indicating that *GH_D07G0638* positively contributes to resistance in this genetic background. In striking contrast, silencing the same gene in the susceptible parent XLZ 4 markedly enhanced resistance, reducing the disease index from 92.5% (control) to 50.0% at 28 dpi. This opposing phenotype suggests that *GH_D07G0638* may function as a susceptibility factor in the susceptible genotype, a phenomenon well documented for several plant – pathogen interactions.

## Discussion

The identification of QTLs associated with VW resistance in upland cotton is challenging due to the lack of immune or highly resistant germplasm and the low polymorphism observed among upland cotton varieties. *Gossypium barbadense* are known to exhibit high levels of resistance to VW [11–13]. To date, most VW resistance QTLs identified have been derived from island cotton. Consequently, significant efforts have been undertaken to map VW resistance loci for introgression into upland cotton to enhance its resistance. The realization of this objective is challenging due to the linkage drag between resistance traits and undesirable agronomic characteristics, as well as the distortion in the segregation of interspecific hybrids [2, 10]. In recent years, cotton breeders have developed and screened some VW resistant upland cotton germplasms that can withstand production testing in China. The mapping VW resistant loci using these germplasms will facilitate VW resistant breeding. Meanwhile, this purpose is further supported by the completion of cotton genome sequencing and ongoing advancements in biotechnology. This study successfully identified and preliminarily validated key VW resistance loci and candidate genes associated with resistance to VW in the upland cotton genome by integrating techniques such as BSA-seq analysis, QTL mapping, transcriptomics, and virus-mediated gene silencing (VIGS), and established a solid foundation for understanding of the molecular mechanisms for upland cotton VW resistance.

Previous studies have mapped clusters of VW-resistance loci on chromosomes A05 and D07. To date, at least 193 quantitative trait loci (QTLs) have been reported, except for chromosomes 10 and 18, which lack any reported VW resistance QTLs. Among these, 111 VW resistance QTLs are situated within VW resistance hotspots across nine chromosomes, as determined by a meta-analysis of QTLs utilizing a *G. hirsutum* × *G. barbadense* population [10]. Previously, researchers identified at least 12 and 21 QTLs associated with VW resistance on Chromosomes A05 and D07, respectively. These QTLs were linked to markers CIR067, CIR224, CIR102, CIR280, CIR062, CIR373, BNL3995, and JESPR065 on Chromosome A05, and A1826, pAR544, HAU0966, BNL1604, BNL1122, JESPR228, HAU2432, and BNL2986 on Chromosome D07. Through meta-analysis, at least one resistance QTL hotspot was detected on Chromosome A05, associated with markers CIR373/BNL3995/JESPR065. Additionally, three resistance QTL hotspots were identified on Chromosome D07, located at 0–20, 30–40 and 50–65 cM, containing 8, 6 and 6 VW resistance QTLs, respectively. These hotspots were linked to markers A1826/pAR544, HAU0966/BNL1604/BNL1122, and JESPR228/HAU2432/BNL2986, respectively [10]. Further basic local alignment searches of the markers associated with VW resistance QTLs on Chromosomes A05 and D07 against the complete genome sequence of *G. hirsutum* revealed that marker BNL2986, linked to the resistance QTL hotspot on Chromosome D07, is located adjacent to qVW-D07. In contrast, the neighboring position of qVW-A05 does not coincide with the VW resistance hotspot on Chromosome A05; however, it encompasses at least three reported VW resistance QTLs [10, 30].

Previous studies have also demonstrated that a greater number of VW resistance QTLs identified on chromosome A05 were derived from the *G. hirsutum* × *G. barbadense* population, whereas the QTLs for VW resistance detected on chromosome D07 originated from *G. hirsutum* × *G. hirsutum* populations. Zhang et al. (2015) [10] used an interspecific Upland × Pima population, Wang et al. (2014) [8] used an interspecific chromosome segment introgression lines population with *G. barbadense* cv Hai7124 as the donor parent, Palanga et al. (2017) [5] used an interspecific backcross population of *Gossypium hirsutum* × *Gossypium barbadense* cv Hai1 respectively for QTL mapping, only one QTL which just explained 1.8% of the phenotypic variation was detected on chromosome D07 by Wang et al. (2014) [8]. In contrast, nine VW resistance QTLs were found on the A05 chromosome. A total of 15 QTLs associated with VW resistance were detected on chromosome D07 through a comparative meta-analysis of *G. hirsutum* × *G. hirsutum* populations [31]. Utilized F_2:3_ population derived from the cross between *G. hirsutum* cv. 60,182 and *G. hirsutum* cv. Junmian 1, four VW resistance QTLs targeting *V. dahliae* isolate BP2, five for *V. dahliae* isolate VD8, and four for *V. dahliae* isolate T9 were identified on chromosome D07 [32]. Furthermore, a genome-wide association analysis involving 290 upland cotton germplasm accessions revealed 10 stable QTLs associated with VW resistance across multiple environments [32, 33]. Notably, one of these QTLs was mapped on chromosome D07, while none were identified on chromosome A05. The pyramid of these 10 elite alleles, each exhibiting the most significant *p-value* for each QTL, reduced the DI of cotton to *V. dahliae* from 70 to 20. Elite alleles *Lsnp1*, *Lsnp4*, *Lsnp5*, *Lsnp8*, and *Lsnp9* have demonstrated a significant increased utilization in contribution to the improvement of VW resistance in Chinese cotton breeding since the 1990s.

The KASP markers tightly linked to qVW-A05 (A05_2289105) and qVW-D07 (D07_6120192 and D07_6896174) are immediately applicable for MAS. Notably, qVW-D07 resides within a 0.74 Mb interval that overlaps with a genomic region harboring the previously reported elite allele *Lsnp8*, which explains up to 11.8% of phenotypic variation. Pyramiding qVW-D07 with *Lsnp8*—or with other favorable alleles on chromosome D07—via multiplex KASP assays may produce additive resistance effects and should be prioritized in breeding programs. The QTL mapping in this study was performed using the *V. dahliae* isolate VD13. Notably, the resistant parent JM122 exhibited high levels of resistance against multiple isolates, including Bp2, V991, and VD13 (Fig. 1), suggesting that its resistance mechanism may be broad-spectrum in nature. Future studies employing mapping populations inoculated with different *V. dahliae* isolates will be necessary to directly validate the contribution of qVW-A05 and qVW-D07 to resistance against various pathotypes, thereby further clarifying their potential in breeding applications. Furthermore, the VW resistance locus qVW-A05 may have been introgressed into JM122, the resistant parent used in this research, from *Gossypium barbadense*. In contrast, the VW resistance locus qVW-D07 originates from resistant upland cotton germplasm and plays a crucial role in enhancing resistance to VW in upland cotton. Importantly, this association analysis was based on VW resistance phenotypes evaluated under natural field conditions in a disease nursery over two consecutive years, providing direct evidence that the markers linked to qVW-A05 and qVW-D07 are effective in predicting resistance under realistic production environments. These findings strongly support the applicability of these QTLs and their linked markers in marker-assisted selection for VW resistance breeding.

In this study, we identified four genes associated with upland cotton VW resistance through VIGS analysis within two QTL intervals. These genes are *GH_A05G0294*, *GH_A05G0389*, *GH_D07G0638*, and *GH_D07G0616*, which encode Atypical CYS HIS rich thioredoxin 1 (*TRX1*), Ferredoxin-like protein (*Fd-like*), Peptide methionine sulfoxide reductase 1 (*MSR1*), and Calcium sensing receptor (*CaSR*), respectively. The *Fd-like* protein is a non-heme iron protein present in bacteria and plants, frequently involved in redox reactions and playing a crucial role in electron transfer processes [34, 35]. *MSR1* and *TRX1* are enzymes that repair oxidized methionine residues in proteins [36–42]. Previous research has demonstrated that TRX1, MSR1, and Fd-like proteins contribute to the regulation of plant growth, development, and stress resistance by inducing reactive oxygen species (ROS) [35, 43–47].Our results showed that the silenced lines of *GhTRX1*, *GhMSR1*, and *GhFd-like* exhibit significant reduction in resistance to VW, further confirming that reactive oxygen species (ROS) homeostasis plays a crucial role in cotton VW resistance [35, 48].

Resistance genes play essential roles in plant disease resistance. However, these genes are not entirely “positive assets”. Sometimes, they may exhibit dual functions, and their function is highly dependent on genetic background and ecological environment. For instance, when resistance genes mutate and lose their ability to trigger immunity, their protein structure can still bind to the effectors of pathogens, playing the role of a “false sentinel” to help pathogens suppress immune responses [49]. The calcium-sensing receptor (*CaSR*), a protein located in the chloroplast thylakoid membrane, is involved in the process of external Ca^2+^-induced increases in cytosolic Ca^2+^ levels in plants. It primarily participates in cell signal transduction, regulation of stress resistance, and maintenance of physiological functions. Furthermore, some research results indicated that the synthesis of salicylic acid (SA) in plants is dependent on *CaSR* [50, 51]. CaSR proteins generally contribute positively to plant disease resistance. Nevertheless, some studies indicate that these proteins serve as key targets for pathogenic effector factors during pathogen attacks. Studies have demonstrated that SsITL inhibits salicylic acid (SA) accumulation in the early stages of infection by interacting with AtCaSR, thereby facilitating infection by *S. sclerotiorum* [52]. Additionally, the effector HopAU1 from *Pseudomonas syringae* pv. *actinidiae* (Psa) interacts with a calcium-sensing receptor in *Nicotiana benthamiana* (NbCaS). Notably, silencing *NbCAS* through RNA interference (RNAi) in *N. benthamiana* significantly reduced HopAU1-triggered cell death, indicating that NbCaS is a critical component for the detection of HopAU1 [53]. In this study, VIGS assays revealed that the *GhCAS* gene plays a positive role in resistant materials, whereas it acts as a susceptibility factor in susceptible materials. We therefore hypothesize that in susceptible upland cotton varieties, *GhCAS* may be hijacked by *V. dahliae* effectors to facilitate infection. This hypothesis warrants further investigation through protein–protein interaction assays and transgenic complementation. Importantly, our findings do not overinterpret *GhCAS* as a universal resistance gene; rather, they highlight its context-dependent role and open new avenues for breeding through S-gene disruption.

## Conclusions

In this investigation, an F_2_ segregating population was derived from a cross between the resistant parent JM122 and the susceptible parent XLZ 4 to map VW resistance QTLs. Through joint analysis of QTL mapping and BSA-seq in two independent repeated identification trials, two stable VW resistance quantitative trait loci, qVW-A05 and qVW-D07, were pinpointed on cotton chromosomes A05 and D07, respectively, accounting for an average of 8.5% and 12.3% of phenotypic variations. Comparative analysis revealed that qVW-A05 and qVW-D07 coincided with known hotspots of cotton resistance to VW. Association analysis results demonstrated that upland cotton germplasms harboring the genotype of the resistant parent JM122 exhibited markedly higher VW resistance compared to those with the genotype of susceptible parent XLZ 4 in natural populations. This suggests that the linkage markers associated with qVW-A05 and qVW-D07 could be utilized for enhancing cotton VW resistance through molecular marker-assisted selection. Expression profiles and VIGS analysis unveiled four potential candidate genes, *GhTRX1*, *GhMSR1*, *GhFd*-like, and *GhCAS*, which may play crucial roles in VW resistance, with *GhCAS* potentially representing a novel and pivotal susceptibility factor for *V. dahliae* infection in susceptible varieties. These findings establish a robust basis for the cloning and characterization of resistance genes in upland cotton, offering valuable insights for future VW-resistance breeding endeavors.

## Supplementary Information

Supplementary Material 1.

## Data Availability

All data used in the current study are included in this published article or are available from the corresponding author on reasonable request.

## Abbreviations

QTL: Quantitative trait locus
KASP: Kompetitive allele-specific PCR
SNP: Single Nucleotide Polymorphism
InDel: Insertion-Deletion
BSA: Bulked Segregant Analysis
VIGS: Virus-induced gene silencing
CAS: Calcium-sensing receptor
DI: Disease index
DR: Death rate
ID: Incidence of disease
