# Supplementary figures and images for: The identification of QTLs and candidate genes associated with VW resistance of Gossypium hirsutum utilizing BSA-seq and QTL mapping
Source: BMC Plant Biol. 2026 May 7;26:1079. doi: 10.1186/s12870-026-08833-y (PMC13295238; doi:10.1186/s12870-026-08833-y)

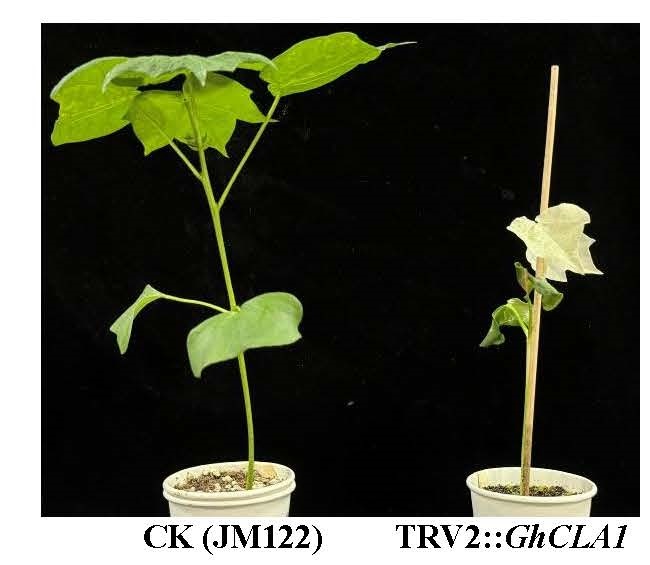

Supplement: Supplementary file 1 — Supplementary Material 1. [file 12870_2026_8833_MOESM1_ESM.zip › Supplementary Figure 2.jpg]

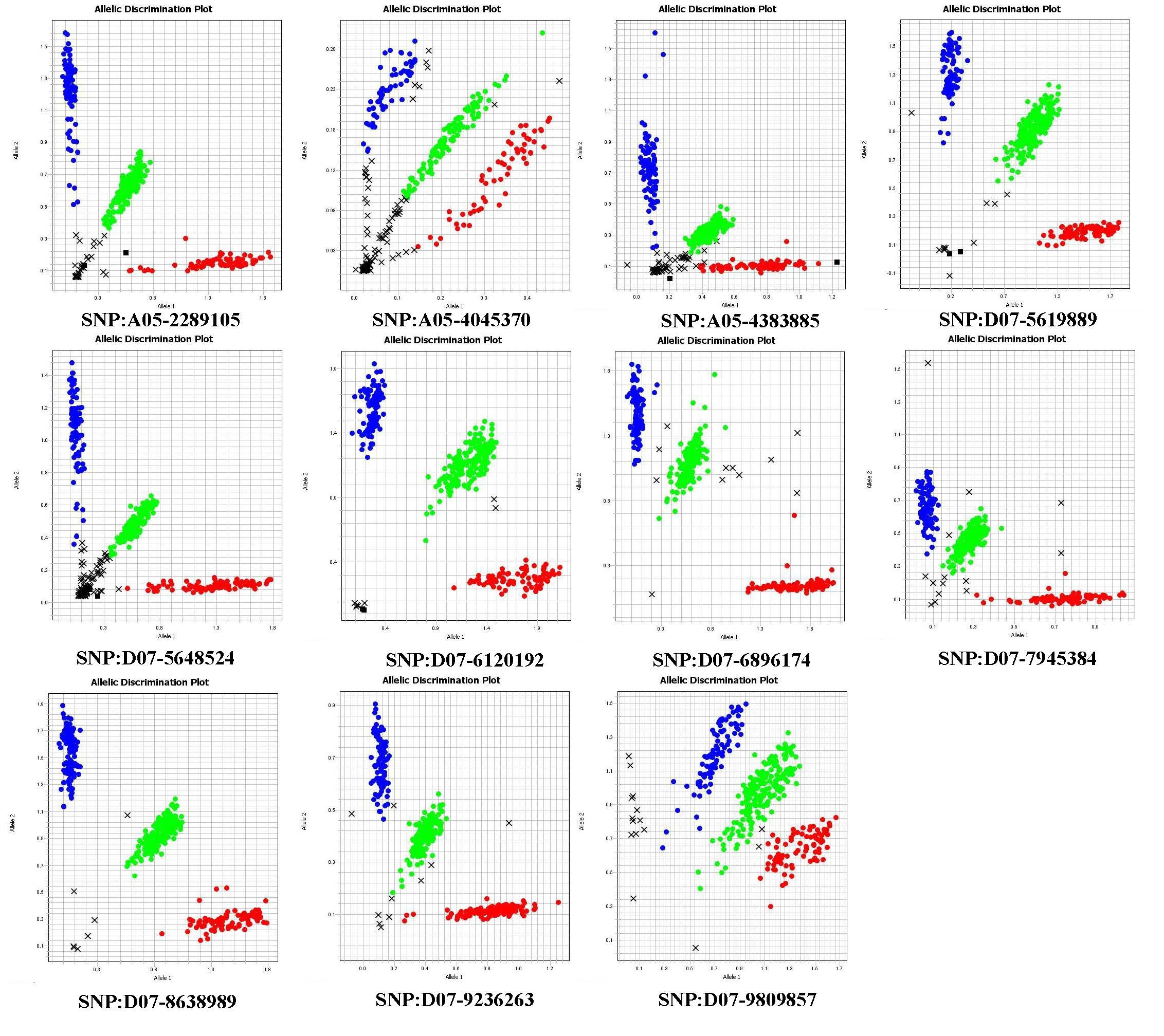

Supplement: Supplementary file 1 — Supplementary Material 1. [file 12870_2026_8833_MOESM1_ESM.zip › Supplementary Figure 1.jpg]
